# Retraction: Upregulation of PTEN in Glioma Cells by Cord Blood Mesenchymal Stem Cells Inhibits Migration via Downregulation of the PI3K/Akt Pathway

**DOI:** 10.1371/journal.pone.0231283

**Published:** 2020-03-30

**Authors:** The PLOS ONE Editors

After this article [1] was published, concerns were raised about results reported in Figures 1, 2, and 5.

Specifically:

- PTEN and pAkt^Ser473^ blots appear the same in the left and right panels of Figure 1B, where the data are reported as representing different experiments.
- β-actin data look similar in lanes 1–5 of Figure 1C and in Figure 1E; the data are reported as representing different experiments.
- GAPDH panels appear similar in Figure 2C (left panel), Figure 5A, and Figure 5B. Figures 5A and 5B represent the same experimental conditions but Figure 2C represents a different experiment.
- XIAP and PDGFR panels appear similar in Figure 5C.

The first author provided electronic image data that are available to support the figures mentioned above, but these data did not clarify the issues outlined.

In addition, the article reports a cDNA microarray experiment but does not mention deposition of the microarray data in a public repository as required by the PLOS Data Policy that was in effect when the article was submitted.

The above concerns call into question the reliability of the reported results and whether the article complied with the journal’s editorial policies. In light of these issues, the *PLOS ONE* Editors retract this article.

VRD did not agree with retraction. The other authors either could not be reached or did not respond directly.
